# Boron Removal in Aqueous Solutions Using Adsorption with Sugarcane Bagasse Biochar and Ammonia Nanobubbles

**DOI:** 10.3390/ma17194895

**Published:** 2024-10-06

**Authors:** Lianying Liao, Hao Chen, Chunlin He, Gjergj Dodbiba, Toyohisa Fujita

**Affiliations:** 1 School of Resources, Environment and Materials, Guangxi University, Nanning 530004, China; liaolianying2022@163.com (L.L.); hchen1996@st.gxu.edu.cn (H.C.); 2 College of Chemistry and Chemical Engineering, Guangxi University, Nanning 530004, China; 3 Graduate School of Engineering, The University of Tokyo, Bunkyo 113-8656, Japan

**Keywords:** sugarcane bagasse, biochar, boron, ammonia nanobubble, adsorption, magnesium ion

## Abstract

Boron is a naturally occurring trace chemical element. High concentrations of boron in nature can adversely affect biological systems and cause severe pollution to the ecological environment. We examined a method to effectively remove boron ions from water systems using sugarcane bagasse biochar from agricultural waste with NH_3_ nanobubbles (10% NH_3_ and 90% N_2_). We studied the effects of the boron solution concentration, pH, and adsorption time on the adsorption of boron by the modified biochar. At the same time, the possibility of using magnesium chloride and NH_3_ nanobubbles to enhance the adsorption capacity of the biochar was explored. The carbonization temperature of sugarcane bagasse was investigated using thermogravimetric analysis. It was characterized using XRD, SEM, and BET analysis. The boron adsorption results showed that, under alkaline conditions above pH 9, the adsorption capacity of the positively charged modified biochar was improved under the double-layer effect of magnesium ions and NH_3_ nanobubbles, because the boron existed in the form of negatively charged borate B(OH)_4_^−^ anion groups. Moreover, cations on the NH_3_ nanobubble could adsorb the boron. When the NH_3_ nanobubbles with boron and the modified biochar with boron could coagulate each other, the boron was removed to a significant extent. Extended DLVO theory was adopted to model the interaction between the NH_3_ nanobubble and modified biochar. The boron adsorption capacity was 36 mg/g at room temperature according to a Langmuir adsorption isotherm. The adsorbed boron was investigated using FT-IR and XPS analysis. The ammonia could be removed using zeolite molecular sieves and heating. Boron in an aqueous solution can be removed via adsorption with modified biochar with NH_3_ nanobubbles and MgCl_2_ addition.

## 1. Introduction

As a chemical element and a trace element from group 13 of the periodic table, boron (B) is a black or brown quasi-metal that is present in the environment in solid or liquid form, such as in borates, boric acid, boric oxide, and salts [1,2,3,4,5]. In recent years, there has been a significant increase in the concentrations of boron in surface water and mine wastewater [6], as boron is widely used in numerous industrial applications, including the production of glass, ceramics, cosmetics, pharmaceuticals, capacitors, semiconductors, soaps, and detergents, and in agriculture in the formulation of some pesticides and fertilizers [1,7,8,9,10,11,12].

Boron enters aquatic ecosystems through various channels. Boron is released into the environment through anthropogenic activities such as agriculture, waste management, sewage treatment, and fossil fuel use. Furthermore, boron enters the environment mainly through natural activities, such as the weathering of rocks, the volatilization of boric acid found in seawater, and volcanic activity [5]. Boron is an essential trace element in animals and plants in nature and plays a vital role in plant growth, development, and reproduction and maintaining the rigidity of plant cells when present in low concentrations [13]. Although it is considered an essential micronutrient, it is important to note that high concentrations of boron can adversely affect plant growth and have implications for animal and human life [14,15]. High concentrations of boron in water can exert adverse effects on aquatic organisms, such as effects on plant cell division, photosynthesis, and respiration. It also accumulates in plants, insects, and fish year after year, causing boron pollution [13].

Furthermore, due to human behavior and geothermal activity, boron solutions in surface water and groundwater are found at concentrations of 0.3 to 100 mg/L or higher. Some researchers believe that excess boron can compromise human health and cause various levels of damage to the body’s nervous system [3,4,5,16]. For this reason, the World Health Organization (WHO) guidelines for drinking water quality recommend a boron concentration of 2.4 mg/L as the maximum permissible limit. Contrary to these regulations, large quantities of industrial boron-containing wastewater are discharged into rivers or oceans. Therefore, it is necessary to develop effective methods to remove boron from aqueous solutions, especially salt solutions. To decrease the environmental risks of boron contamination in water bodies, chemical and engineering techniques to remove this contaminant from water have been developed based on membrane technology [17], precipitation [18], electrocoagulation [19,20,21,22], reverse osmosis [23,24,25,26,27], ion exchange [28,29,30], and adsorption [9,31,32,33,34]. Among these methods, adsorption is considered one of the most effective and popular boron removal methods due to its low cost, good absorption effect, high recovery, renewable adsorbent, recyclability, and easy operation.

The adsorption process involves the adsorption of boron on the surface of the adsorbent via chemical or physical action. Therefore, the design of the adsorbent is critical for effective removal. Several boron adsorbents with different physical and chemical structures have been reported. Boron adsorbents include adsorbents functionalized by chelating groups [35,36,37,38], metal-oxide-based adsorbents [39,40,41,42,43,44,45,46], clays, and layered double hydroxides (LDHs) [47,48,49]. Among them, the chelating resin is one of the most mature commercial boron adsorption materials. Boron-chelating resins containing glucosamine groups effectively remove boron from contaminating solutions via cis-hydroxyl chelation [50]. The presence of two ortho hydroxyl groups in the N-methyl-d-glucosamine group allows boric acid and borate to form stable complexes with the adsorbent [34]. However, most boron-chelating resins are produced from synthetic polymers that are difficult to degrade and, therefore, pose environmental concerns [51,52,53]. In addition, the synthesis process of some boron-chelating resins is cumbersome, the adsorption performance still does not meet the requirements, the synthesis process is complex, and the cost is high, which create certain limitations in the practical application field. Therefore, preparing and studying new alternative materials with low costs, good biodegradability, and high adsorption efficiency are necessary. A selective adsorbent is preferable [54,55]; however, boron ions are difficult to adsorb via the methylglucamine-type ion exchange method. Therefore, other heavy metal ions should be removed first through sedimentation and adsorption. Then, the boron ions can be removed using this method.

Sources of raw biochar materials are extensive and abundant, and they are easy to obtain. At the same time, the preparation of biochar is of great significance in transforming waste into valuable products and reducing greenhouse gas emissions. When humans engage in agricultural production activities, they produce a large amount of agricultural waste. A portion of this agricultural waste can be prepared into biochar through heat treatment. Products such as dragon fruit peel, banana peel, grapefruit peel, sugarcane bagasse, etc. contain a large amount of organic matter, such as xylitol, lignin, and cellulose, which are rich in content and have strong usability [56]. They are very valuable products and raw materials that can be used to prepare biochar. Sugarcane bagasse is the most widely used and cost-effective agricultural production waste. Brazil is one of the world’s largest sugarcane-producing countries. During the 2021–2022 harvest period, Brazil produced and processed a total of 585.2 million tons of sugarcane, producing 35 million tons of sugar and 23.3 million cubic meters of ethanol, equivalent to 59.9 kg of sugar and 39.8 L of ethanol per ton of sugarcane [57]. This sugarcane leaves a large amount of bagasse waste after processing. In addition, Guangxi is one of China’s largest sugarcane production areas, and there is a large amount of waste every year [58]. Therefore, it is necessary to recycle sugarcane and prepare biochar from sugarcane waste. Biochar produced from sugarcane bagasse is rich in carbon, with fine particles and a porous structure, and is commonly used in wastewater treatment [59]. Sugarcane bagasse is rich in lignin, hemi-fiber, and cellulose [60], making it beneficial in removing boron elements from aqueous environments. Sugarcane bagasse has adsorption properties, but, due to the lack of surface functional groups on the surface of pure sugarcane bagasse, it cannot interact well with borate ions, and the adsorption effect on boron is insignificant. Therefore, it is necessary to carry out composite modification on pure sugarcane bagasse. The formation of new surface functional groups increases the adsorption of boron by sugarcane bagasse. Different modification reagents will result in different modified sugarcane bagasse products, and their adsorption effects will also vary.

Nanobubbles are most commonly defined as nanoscale gas cavities with a diameter of less than 1 μm. Therefore, nanobubbles have a large specific surface area. Nanobubbles are represented by two different types of bubbles in liquids: surface and bulk bubbles. In this experiment, bulk nanobubbles are employed. The nanobubble and nanobubble or solid particle interactions can be determined using the extended Derjaguin–Landau–Verwey–Overbeek (DLVO) theory [61]. Here, in order to control the zeta potential of the adsorbents and nanobubbles, MgCl_2_ was added. Next, modified biochar with Mg^2+^ ions and NH_3_ nanobubbles that could interact with Mg^2+^ ions were investigated regarding their coagulation in a boron-containing solution to remove boron from an aqueous solution. According to the drinking water quality standards [62], the amount of Mg (and Ca) should be less than 100 ppm for the taste threshold, that of NH_3_ should be less than 35 ppm for the taste threshold, and that of Cl should be 5 ppm for the smell threshold in drinking water. In this study, these water quality standards were considered.

## 2. Materials and Methods

### 2.1. Materials

The sugarcane bagasse used to prepare the adsorbent for the experiment was purchased in Nanning City, Guangxi Province. All chemicals (NaOH, HNO_3_, H_3_BO_3_, MgCl_2_, CaCl_2_, N, N-dimethylformamide (DMF), epichlorohydrin (ECH), ethylenediamine (EDA), triethylamine (TEA)) were purchased from Aladdin Biochemical Technology Co., Ltd. (Shanghai, China) and were of analytical grade. The solutions were all prepared with deionized water (18.2 MΩ∙cm).

**Figure 1:**
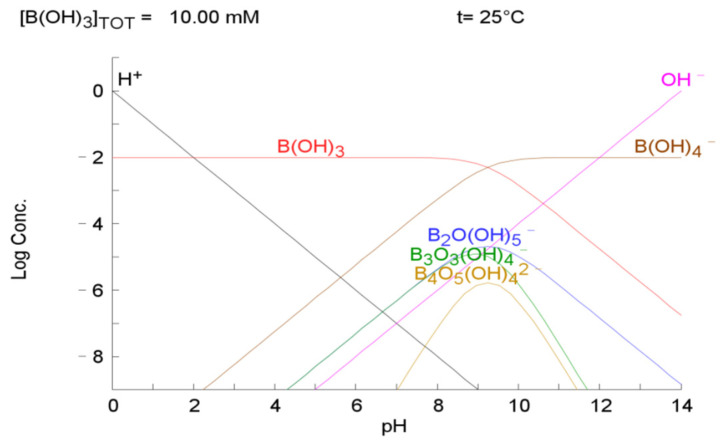
Speciation diagram for boron.

The morphological distribution of boron in an aqueous solution in 10 mM B(OH)_3_ is shown in Figure 1, which was obtained through a chemical equilibrium diagram (software, Eq-Diagr-Java 7, Chemical Equilibrium Calculations software). Boron exists in the form of negative ion groups and consists mainly of B(OH)_4_^−^ ions in an alkaline region of more than pH 9.

H_3_BO_3_(aq) + H_2_O(l) → H^+^ + B(OH)_4_^−^(aq), p*K*a = 9.24 (1)

### 2.2. Adsorbent Preparation Methods

#### 2.2.1. Preparation Method for Char A

The sugarcane bagasse was obtained from Chaoyang Square, Nanning, Guangxi and was cleaned three times with deionized water to remove as the impurities on the surface, followed by storage in a constant-temperature drying box at 80 °C for 24 h until the moisture in the biomass was fully evaporated to dryness. The dried sugarcane bagasse was crushed with a crusher, passed through a 20-mesh screen, and stored in a sealed bag for further use.

**Figure 2:**
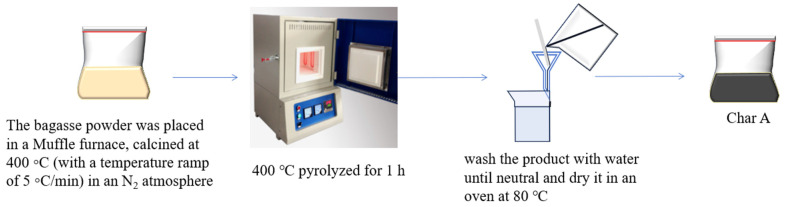
Preparation method of char A.

The preparation method for biochar using sugarcane bagasse is shown in Figure 2. The dried sugarcane bagasse powder was placed in a muffle furnace, calcined at 400 °C (with a temperature ramp of 10 °C/min) in an N_2_ atmosphere, and pyrolyzed for 1 h, after which it was washed and dried to obtain char A.

#### 2.2.2. Preparation Method for Char B, B1, B2, and B3

**Figure 3:**
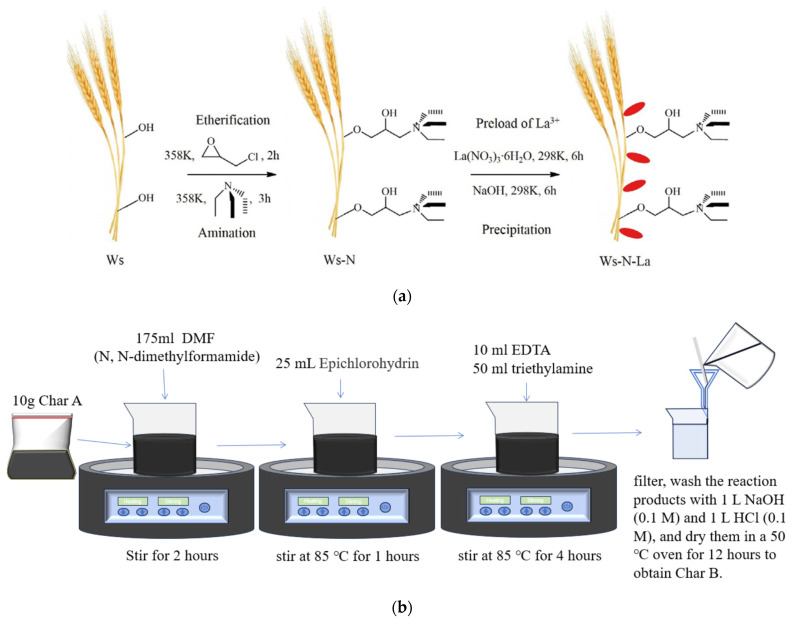
Preparation method. (**a**) Schematic procedure for the preparation of nanocomposite Ws–N–La [63]. (**b**) Preparation method of char B.

Figure 3 shows the preparation method for char B. First, 10 g of dried char A was mixed with 175 mL of N,N-dimethylformamide (DMF) at ambient temperature for 2 h; then, 25 mL epichlorohydrin was added to the mixture as an agent and it was stirred for etherification for 1 h at 85 °C, followed by the slow addition of 10 mL of EDTA and 50 mL of triethylamine to graft quaternary ammonium groups on char A. The solution was stirred for 4 h at 85 °C and then filtered, and the reaction product was washed with 1 L of NaOH (0.1 M), 1 L of HCl (0.1 M), and 1 L of 50% ethanol solution and then dried in an oven at 50 °C for 12 h to obtain char B. The reaction is shown in Figure 3a [63].

On the other hand, each reagent was used to prepare modified char A to investigate the adsorption effect of the reagent. First, 10 g of dried char A was mixed with 200 mL of N,N-dimethylformamide (DMF) in an 85 °C water bath and stirred for 5 h. Then, it was filtered, washed, and dried using the same method shown in Figure 3 to obtain char B1.

Then, 10 g of dried char A was mixed with 200 mL of EDTA in an 85 °C water bath and stirred for 5 h; 10 g of dried char A was mixed with 200 mL of triethylamine in an 85 °C water bath and stirred for 5 h; next, it was filtered, washed, and dried using the same method shown in Figure 3 to obtain char B3.

### 2.3. Preparation Method for NH_3_ with N_2_ Nanobubbles

**Figure 4:**
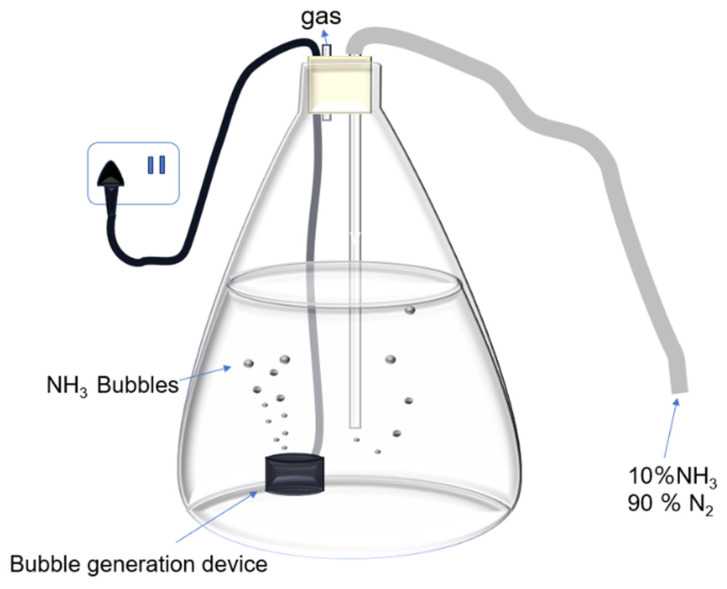
Preparation method for NH_3_ and N_2_ nanobubbles, using ultrasonic atomization equipment.

The preparation method for NH_3_ with N_2_ nanobubbles is shown in Figure 4. The nanobubbles were prepared in 1 L of deionized water in a 2 L conical flask, which was placed in an ultrasonic atomization equipment bubble generator (Dongguan Run Yang Electronic Co., Ltd., Dongguan, China) and a gas flow device. A mixture of 10% NH_3_ and 90% N_2_ was introduced as the gas. After five minutes of aeration, the bubble generator was turned on to prepare the bubbles, during which the gas was continuously aired. After 20 min, the valve and bubble generator were closed. Then, an aqueous solution with ammonia nanobubbles could be collected in the water. The ammonia gas reaction is shown in the following Equations (2) and (3) [64].

NH_3_(g) ⇌ NH_3_(aq), NH_3_(aq) + H_2_O(l) ⇌ NH_4_^+^(aq) + OH^−^(aq), (2)

*K*_b_ = [NH_4_^+^][OH^−^]/[NH_3_] = 1.75 × 10^−5^ at 18 °C

NH_4_^+^ + H_2_O ⇌ NH_3_·H_2_O + H^+^ (3)

*Ka* = [NH_3_·H_2_O][H^+^]/[NH_4_^+^] = 5.88 × 10^−10^ at 25 °C

The NH_4_^+^ ion concentration in the NH_3_ nanobubble solution was 1900 ppm in this experiment. Moreover, water containing only nitrogen nanobubbles could be prepared using the same generator with 100% nitrogen gas.

### 2.4. Analysis Methods

Thermogravimetry–differential thermal analysis (TG-DTA; NETZSCH, STA 449F3, Jupiter, Selb, Germany) was used to investigate the thermal stability of sugarcane bagasse under a nitrogen atmosphere. X-ray photoelectron spectroscopy (XPS; Thermo, K-Alpha+, Scientific, Nexsa, USA, C 1s: 284.6 eV) was adopted to analyze the chemical states of the elements. The boron content in the samples was tested with an inductively coupled plasma atomic emission spectrometer (ICP-AES; ICPS 7510, Shimadzu, Concept, Japan). The surface charge of the biochar was measured with a zeta potential analyzer (NanoBrook Omni, Brookhaven, Holtsville, NY, USA). A new high-resolution field emission scanning electron microscope (SEM; SU8020, Hitachi, Limited, Tokyo, Japan) was used to observe the surface morphologies of all biochar samples. The biochar was analyzed via Fourier transform infrared spectroscopy (FT-IR; Shimadzu IRTracer-100, Shimadzu, Kyoto, Japan) using a pressed KBr disc. The specific surface area was measured via Brunauer–Emmett–Teller (BET) analysis, and the porosity of the samples was assessed via N_2_ adsorption–desorption experiments using the Barrett, Joyner, and Halenda (BJH) method (Ttistarll3020, Micromeritics, Norcross, GA, USA). Nanoparticle tracking analysis (NTA; NanoSight NS300 device, Malvern Panalytical Ltd., Malvern, UK) was used for nanobubble size characterization and determination. The powder X-ray diffraction (XRD) patterns were measured on an X’Pert PRO diffractometer with Cu K radiation (λ = 0.154 nm).

### 2.5. Adsorption Experiments

The boronic acid solution utilized for adsorption in the experiment was prepared by weighing a specific quantity of boronic acid and transferring it into a volumetric flask. Distilled water was added to dissolve the boronic acid, resulting in the desired concentration of the aqueous boronic acid solution.

The efficacy and underlying principles of the char adsorbent were meticulously examined, considering many factors, such as the pH, temperature, contact time, solution concentration, and adsorbent dosage. Aqueous solutions of boric acid were used to simulate the presence of boron in water. A constant amount of 25 mL of boric acid solution was used throughout these adsorption experiments.

In the adsorption experiment, the aqueous boric acid solution contained 200 mg·L^−1^ of boron. The adsorption experiment was carried out by mixing 10 mg biochar with 25 mL (200 mg·L^−1^ B(OH)_4_^−^ solution) in a polyethylene bottle and shaking at a constant temperature (298 K) in a water bath with a velocity of 140 rpm. Each experiment was repeated three times to ensure the reliability of the experimental data. The adsorbent was separated from the liquid using a suction filter (0.45 μm microporous membrane). The quantification of boron elements in the solution post-adsorption was achieved using ICP-AES, which facilitated the computation of the adsorption capacity on a per-boron-element basis. The adsorption capacity of the adsorbent (*q_e_*, mg·g^−1^) was calculated using Equation (4).

(4) $$q_{e}=\frac{\left(C_{0}-C_{e}\right)*V}{m}$$

where *q_e_* (mg·g^−1^) represents the amount of adsorption at equilibrium, *C_e_* (mg·L^−1^) is the equilibrium concentration of the solution, *C*_0_ (mg·L^−1^) is the initial concentration of the solution, *V* (L) is the volume of the solution, and *m* (g) is the mass of the adsorbent used.

## 3. Results and Discussion

### 3.1. Material Characterization

#### 3.1.1. Thermogravimetric Analysis

**Figure 5:**
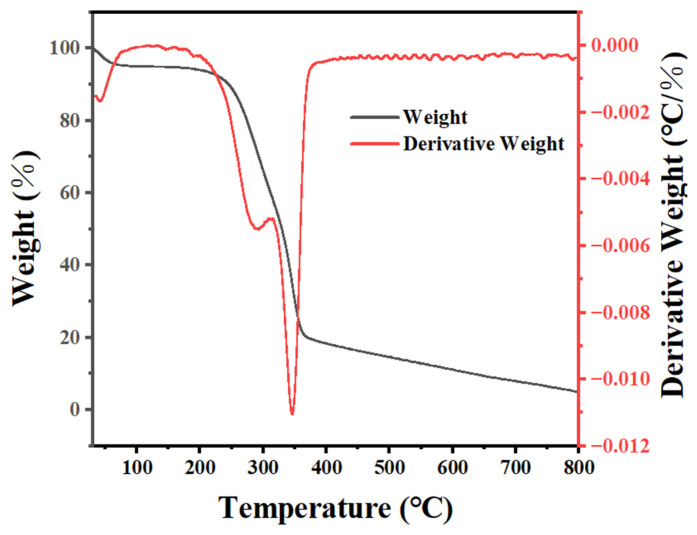
TGA and DTG curves of sugarcane bagasse under N_2_ air atmosphere at a heating rate of 5 °C min^−1^.

Thermogravimetric analysis (TGA) was performed to confirm the temperature of biochar preparation. The TGA and DTG curves of sugarcane bagasse under a N_2_ air atmosphere at a heating rate of 5 °C min^−1^ are shown in Figure 5. The TGA curve (the black line in Figure 5) of sugarcane bagasse showed the first weight loss interval between 30 °C and 150 °C, which might have resulted from the vaporization of water and other volatile compounds. Meanwhile, between 150 °C and 364 °C, another region of weight loss was observed, which resulted from the expulsion of organic compounds such as fats, waxes, alkaloids, and terpenes. In addition to these two losses, a third region of weight loss was associated with the thermal degradation of lignin, hemicellulose, and cellulose, and the decarboxylation reactions formed carbon, ketones, and aldehydes, occurring between 364 °C and 800 °C [65,66,67]. The DTG curve (the first derivative of TGA, the red line in Figure 5) of sugarcane bagasse showed a peak at 346 °C, corresponding to the maximum rate of mass loss related to the thermal decomposition of cellulose. A shoulder at 293 °C was associated with the thermal decomposition of the hemicellulose [68]. In this study, a temperature of 400 °C was used to prepare char with sugarcane bagasse.

#### 3.1.2. XRD Analysis

**Figure 6:**
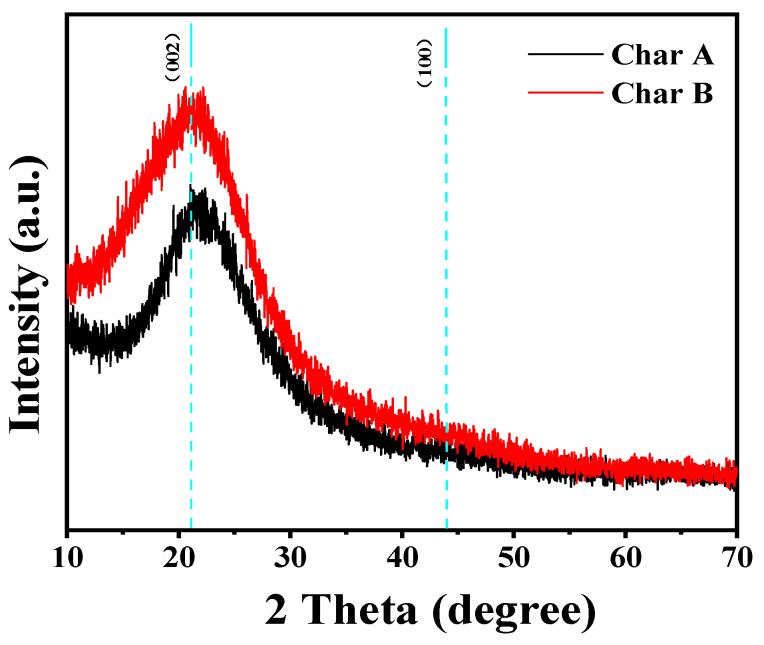
XRD patterns of char A and char B.

The X-ray diffraction spectra obtained for char A and char B are shown in Figure 6. The broad characteristic peak at 2 theta (θ) = 21.9° was ascribed to the (002) plane of graphite microcrystals in amorphous carbon. For char B, the (002) peak moved towards more left 2θ angles than char A. This result indicated that favorable defects could be formed in the prepared char B. On the other hand, the peak at 2θ = 43.8° is considered the (100) plane of the graphitic structure [69,70,71,72]. However, the (100) peak did not appear in the XRD patterns for chars A and B. The chars A and char B prepared at 400 °C did not reach the crystallization temperature of the graphitic structure.

#### 3.1.3. SEM Analysis

**Figure 7:**
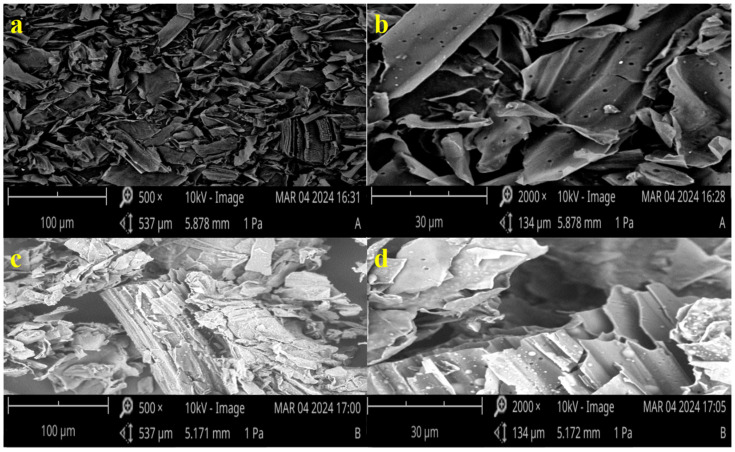
SEM photographs of char A and char B. Char A: magnitude of 500× (**a**) and magnitude of 2000× (**b**). Char B: magnitude of 500× (**c**) and magnitude of 2000× (**d**).

SEM analysis was conducted to determine the surface morphologies of char A and char B. The SEM micrographs of char A are shown in Figure 7a,b and those of char B are shown in Figure 7c,d. Char A can be observed to possess heterogeneous, amorphous, and irregular surfaces in Figure 7a, and a few pores are seen in Figure 7b. When comparing char A and char B, it was found that the morphologies slightly changed. Char B presented a porous structure with abundant pores and showed numerous inner-pore channel structures with smooth inner-pore walls and an increased size.

#### 3.1.4. Specific Surface Area and Porosity

**Figure 8:**
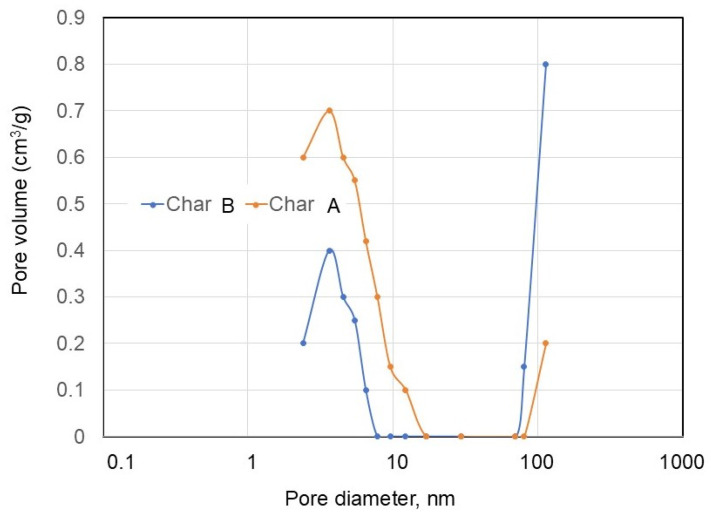
Pore size distribution depending on pore diameter.

The surface areas of char A and char B were analyzed based on the N_2_ adsorption–desorption isotherms obtained using multipoint BET analysis, and the total pore volume and pore diameter were determined using the BJH method. They are listed in Table 1, and the pore size distribution depending on the pore diameter is shown in Figure 8. It was observed that char A had a surface area of 42.56 m^2^/g, and char B had a surface area of 1.59 m^2^/g. Char A’s surface area was larger than that of char B. However, when comparing the pore diameters of char A and char B, that of char A was smaller than that of char B. Char A was a microporous material and char B was a mesoporous material [73,74].

#### 3.1.5. Nanoparticle Size and Stability

**Figure 9:**
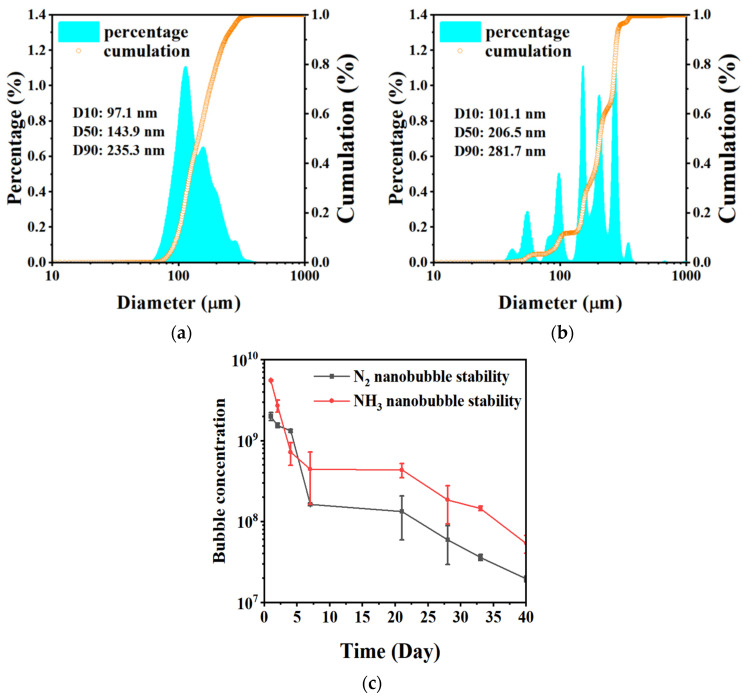
Nanobubble size distributions and bubble concentrations of NH_3_ and N_2_ nanobubbles. (**a**) Size distribution of NH_3_ nanobubbles (10% NH_3_ and 90% N_2_, pH 11.2), (**b**) size distribution of N_2_ nanobubbles (100% N_2_, pH 10.4), (**c**) changes in concentrations of NH_3_ nanobubbles and N_2_ nanobubbles over time.

The particle concentration and size were determined using NTA at room temperature. The test was conducted within one hour of the completion of bubble preparation. The size distributions of the NH_3_ nanobubbles (10% NH_3_ and 90% N_2_) at pH 11.2 and the N_2_ nanobubbles (100% N_2_) at pH 10.4 are illustrated in Figure 9a and Figure 9b, respectively. The uniformity coefficient d (60%)/d (10%) was 1.7 for NH_3_ nanobubbles. The uniformity coefficient d (60%)/d (10%) was 2.2 for N_2_ nanobubbles. The NH_3_ nanobubble particle size was more uniform than that of N_2_ nanobubbles.

The changes in the concentrations of NH_3_ nanobubbles at pH 11.2 and N_2_ nanobubbles at pH 10.4, depending on time, are shown in Figure 9c. The particle size distributions shown in Figure 9a,b were measured on the initial preparation day, and the particle concentrations were 5.6 × 10^9^ particles/mL for NH_3_ nanobubbles and 2.0 × 10^9^ particles/mL for N_2_ nanobubbles. Both particle concentrations decreased rapidly over a period of five days and gradually decreased after the fifth day, as shown in Figure 9c. During this period, the particle concentration of NH_3_ nanobubbles was higher than that of N_2_ nanobubbles almost every time. As the nanobubbles have a large specific surface area, there is the possibility for a large amount of ion adsorption under optimal conditions.

#### 3.1.6. Zeta Potential of Char and Nanobubbles

**Figure 10:**
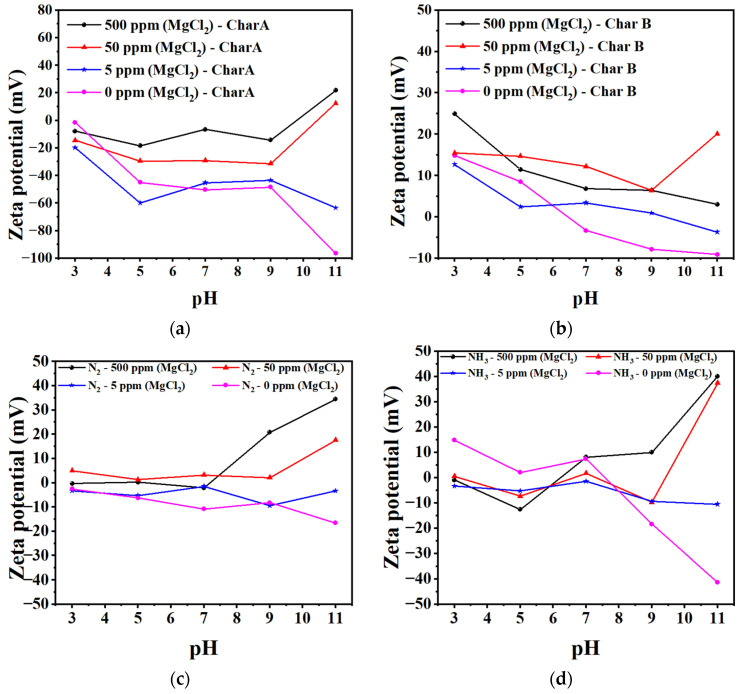
Zeta potential as a function of pH for adsorbents and nanobubbles. Zeta potential in MgCl_2_ solutions of different concentrations of char A (**a**); char B (**b**). Zeta potential in MgCl_2_ solutions of different concentrations of N_2_ nanobubbles (**c**) and NH_3_ nanobubbles (**d**).

In order to study the potential of the adsorbent, a zeta potential analysis was conducted. The zeta potentials of different concentrations of char A and char B in MgCl_2_ solutions are shown in Figure 10a and Figure 10b, respectively. As the concentration of the MgCl_2_ solutions increases, the negative zeta potential gradually becomes more positive in the alkaline region. The zeta potentials of char B are more positive than those of char A. Char B could adsorb to negative ions more easily than char A. Under alkaline conditions, boron existed in the form of negative ion groups, as shown in Figure 1; therefore, boron adsorption would be possible on the positive surface.

The zeta potentials of different concentrations of N_2_ nanobubbles and NH_3_ nanobubbles in MgCl_2_ solutions are shown in Figure 10c and Figure 10d, respectively. Both types of nanobubbles showed negative charges in the alkaline region. However, these negative charges became slightly negative in the 5 ppm MgCl_2_ solution. With higher concentrations of the MgCl_2_ solution, the zeta potential became more positive in the alkaline region.

The negative zeta potentials of both the NH_3_ nanobubbles and char became slightly negative to positive when increasing the addition of MgCl_2_ in the alkaline region. NH_4_^+^ ion exists due to the solubility of NH_3_ nanobubbles in the solution, as shown in Equations (2) and (3), and the NH_4_^+^ ions decreased as the alkaline pH increased. Therefore, the NH_3_ nanobubble’s surface was negative in the alkaline region and changed from slightly negative to positive as the MgCl_2_ amount was increased.

#### 3.1.7. Interaction with Char and Nanobubbles

**Figure 11:**
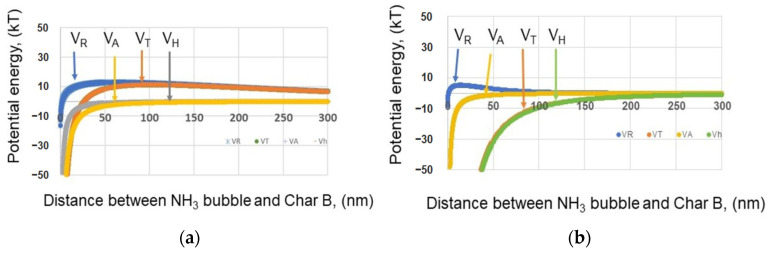
Total potential energy V_T_ as a function of the distance between the NH_3_ nanobubbles and char B at pH 9. (**a**) No addition of MgCl_2_, NH_3_ nanobubbles −18 mV and char B −8 mV; (**b**) addition of 5 ppm MgCl_2_ and 200 ppm B, NH_3_ nanobubbles −5 mV and char B −13 mV. Here, the NH_3_ nanobubble size is 200 nm, and the char B size is 10 mm.

The interaction between the NH_3_ nanobubbles and char B was estimated using the extended DLVO theory [59]. The total potential energy (V_T_), van der Waals interaction energy (V_A_), hydrophobic interaction energy (V_H_), and electrostatic interaction energy (V_E_) between NH_3_ nanobubbles and char B are shown in Figure 11. If there is no addition of MgCl_2_, there is an approximately 10 kT barrier of total potential energy (V_T_) between the NH_3_ nanobubbles and char B, as shown in Figure 11a. The coagulation possibility between NH_3_ nanobubbles and char B is low. On the other hand, when adding 5 ppm MgCl_2_ in 200 ppm B aqueous solution, there is no total potential energy barrier, as shown in Figure 11b. Therefore, the NH_3_ nanobubbles and char B will coagulate easily.

### 3.2. Boron Adsorption

#### 3.2.1. Concept of Boron Adsorption Using Adsorbent and Nanobubbles

**Figure 12:**
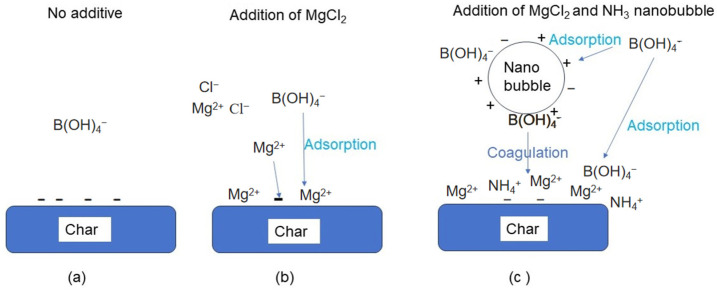
Boron adsorption types. (**a**) No additives, (**b**) addition of MgCl_2_, (**c**) addition of MgCl_2_ and NH_3_ nanobubbles.

The boron adsorption types are shown in Figure 12. If there are no additives in the boron-containing aqueous solution, B(OH)_4_^−^ anions, shown in Figure 1 in an alkaline region, cannot adsorb on the negatively charged char, as shown in Figure 12a. When MgCl_2_ is added to the solution, the negatively charged char adsorbs Mg^2+^ ions, and the surface charge becomes positive. Therefore, B(OH)_4_^−^ can be adsorbed on the positive surface of the char, as shown in Figure 12b. The interactions between the nanobubbles and char when both NH_3_ nanobubbles and MgCl_2_ are added to the solution are shown in Figure 12c. The Mg^2+^ ions are adsorbed on the negative nanobubble surface and char. B(OH)_4_^−^ ions can be adsorbed at the positive site of the NH_3_ nanobubbles and char. In particular, the nanobubble’s specific surface area is very large, and there are a large number of nanobubbles—approximately 10^9^—after a few days, as shown in Figure 9. When the boron-adsorbed NH_3_ nanobubbles and boron-adsorbed char coagulate, a large amount of B(OH)_4_^−^ will be removed from the aqueous solution. As shown in Figure 11b, the B(OH)_4_^−^-adsorbed NH_3_ nanobubbles can coagulate with the B(OH)_4_^−^-adsorbed char B with 5 ppm MgCl_2_ at pH 9, as there is no potential barrier between the nanobubbles and char. An additional 5 ppm MgCl_2_ was employed while considering the drinking water quality standards [60].

#### 3.2.2. Effects of pH, Different Adsorption Amounts, Contact Time, and Temperature

**Figure 13:**
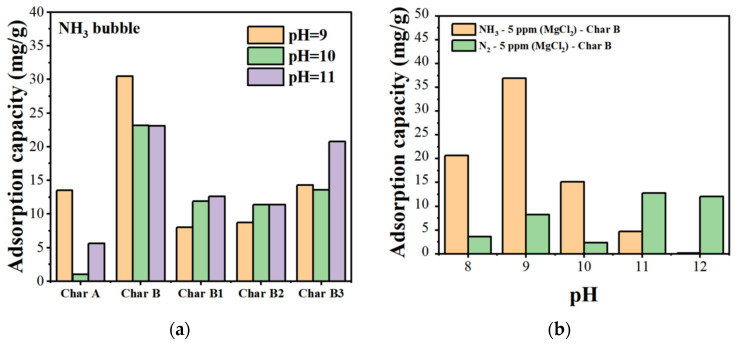

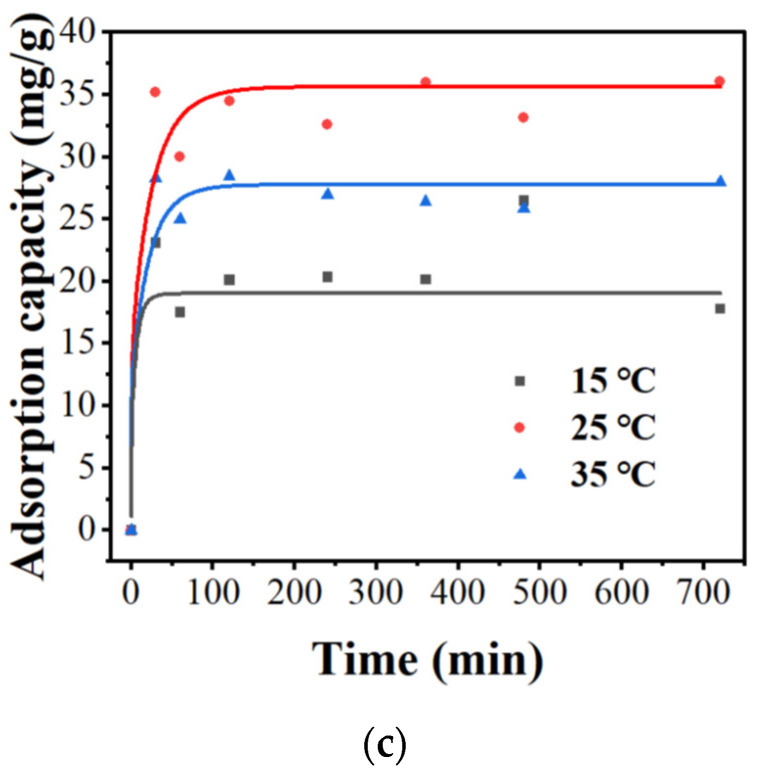
Boron adsorption capacity in different conditions. (**a**) Boron adsorption in different adsorbents with 5 ppm MgCl_2_; (**b**) Comparison of N_2_ and NH_3_ nanobubbles with 5 ppm MgCl_2_ for char B adsorbent. (**c**) Boron adsorption capacity at different temperatures and contact times.

The effect of the pH on the boron adsorption of the adsorbents with 5 ppm of MgCl_2_ aqueous solution is shown in Figure 13a. As the pH rose, the boron adsorption capacity of the adsorbents increased; it peaked at pH 9 and then decreased with a further increase in the pH. Char B–MgCl_2_ (5 ppm)–NH_3_ nanobubbles resulted in the highest boron adsorption capacity and reached a maximum of 35.5 mg/g at pH 9. The zeta potential of char A was negative at −40 to −60 mV from pH 9 to 11 with 5 ppm MgCl_2_ in the solution, as shown in Figure 10a. The B(OH)_4_^−^ ions were difficult to adsorb on the negative surface of char A. Meanwhile, the zeta potential of char B was positive at +3 mV at pH 9 with 5 ppm MgCl_2_ in the solution, as shown in Figure 10b, and the B(OH) _4_^−^ ions could adsorb on the positive surface of char B. When increasing the pH from 9 to 11, the zeta potential of char B became slightly negative, and the boron adsorption amount on char B decreased. The zeta potentials of chars B1, B2, and B3 were slightly negative from pH 9 to 11 with 5 ppm MgCl_2_. The adsorption rate of B(OH)_4_^−^ ions was not high. When the pH changed in the alkaline area, the zeta potential became high with a negative charge and boron desorption became possible, as well as its regeneration.

The comparison of N_2_ and NH_3_ nanobubbles with 5 ppm MgCl_2_ aqueous solution for the char B adsorbent is shown in Figure 13b. The boron adsorption capacity of char B–(5 ppm) MgCl_2_–NH_3_ nanobubbles was the largest at 36 mg/g at pH 9; this value was higher than that for char B–(5 ppm) MgCl_2_–N_2_ nanobubbles, with 8 mg/g at pH 9. The Mg^2+^ ions were adsorbed on the N_2_ and NH_3_ nanobubble surface, and the zeta potential changed from a large negative value to a slightly negative value, as shown in Figure 10c,d, at pH 9 to 11. Boron mainly exists as B(OH)_4_^−^ ions at pH 9 to 11, as shown in Figure 1. B(OH)_4_^−^ can adsorb Mg^2+^ on the surfaces of nanobubbles. The NH_3_ nanobubbles supplied NH_4_^+^ cations mainly by dissolving water, as shown in Equations (2) and (3). The NH_4_^+^ ions adsorbed on the surface of the adsorbent can also be adsorbed by B(OH)_4_^−^ ions as well as Mg^2+^ ions.

We studied the effects of different contact time and temperatures on the adsorption of char B–MgCl_2_ (5 ppm)–NH_3_ nanobubbles. As shown in Figure 13c, the boron uptake increased with time and reached 35 mg/g at the temperature of 25 °C. Compared with the adsorption in an aqueous solution at 15 °C and 35 °C, the adsorption efficiency of boron at 25 °C was improved to 75.0% in the solution.

Table 2 shows a comparison of the boron adsorption capacities among different boron adsorption materials.

#### 3.2.3. Adsorption Isotherm and Adsorption Kinetics Study

**Figure 14:**
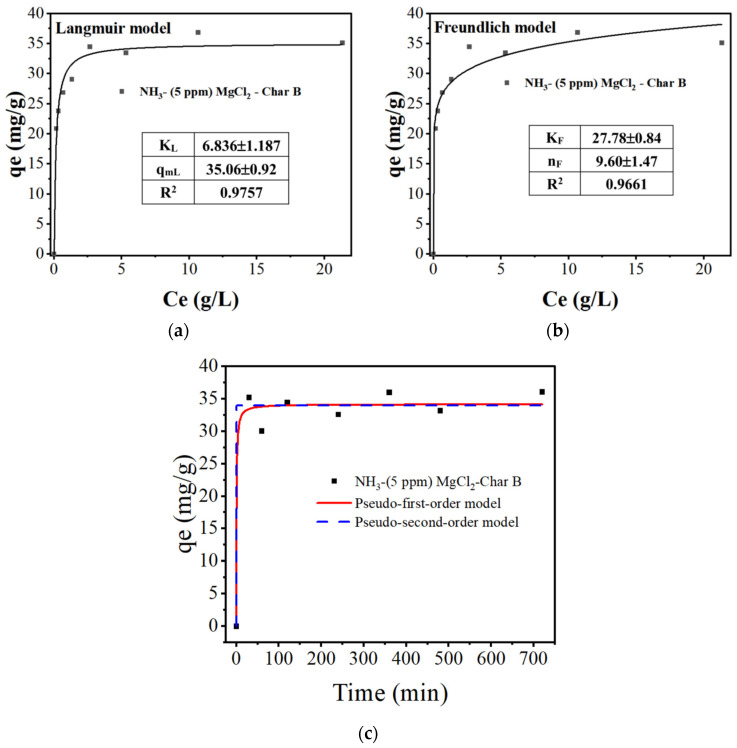
Boron adsorption isotherm and kinetics for char B with 5 ppm MgCl_2_ and NH_3_ nanobubbles at pH 9 (adsorbent mass/volume of solution (m/V) is 0.4 g/L, measured temperature is 298 K, adsorption time is 2 h). (**a**) Langmuir adsorption isotherm, (**b**) Freundlich adsorption isotherm, (**c**) effect of adsorption capacity depending on time.

In order to explore the adsorption mechanism and predict the maximum adsorption capacity, two typical adsorption isotherm models, the Freundlich adsorption isotherm and Langmuir adsorption isotherm, were used to fit and analyze the data, as shown in Figure 14a,b, respectively. The adsorption isotherm was studied for the adsorbent composed of char B with NH_3_ nanobubbles and 5 ppm MgCl_2_ at the optimum pH of 9. The adsorbent mass/volume of solution (m/V) was 0.4 g/L, with a temperature of 298 K, and the shaking time for adsorption was 2 h. It could be seen that the adsorption capacity of boron increased rapidly as the equilibrium concentration in the solution increased, and it quickly reached saturation and a steady state. The adsorption capacity of char B with 5 ppm of MgCl_2_ and NH_3_ nanobubbles was higher than the data reported in other works [9,75,76,77,78,79,80].

The Freundlich isotherm is shown in the following formula [81]:

(5) $$q_{e}=k_{F}C_{e}^{n_{F}}$$

where *q_e_* is the equilibrium adsorption capacity, and *k_F_* and *n_F_* are the adsorption potential and strength constants of the Freundlich isotherm model, respectively.

The Langmuir isotherm is shown in the following formula [81]:

(6) $$q_{e}=\frac{q_{m,L}k_{L}C_{e}}{1\pm k_{L}C_{e}}$$

where *q_m,L_* is the maximum adsorption capacity of the Langmuir isotherm (mg/g), and *k_L_* is the Langmuir isotherm constant or affinity constant (dm^3^/mg).

The fitting curves for the isotherms are shown in Figure 14a,b. The fitting results of the Langmuir and Freundlich isotherm models are listed in the figure. The Langmuir isotherm equation showed a higher correlation coefficient (R^2^ 0.9757) than the Freundlich isotherm equation (R^2^ 0.9661). Boron was adsorbed in a single layer, and the Langmuir equation was used to calculate the theoretical adsorption capacity of boron, resulting in a value of 36 mg/g.

*ln*(*q_e_* − *q_t_*) = *lnq_e_* − *K*_1_*t* (7)

*t*/*q_t_* = 1/(*K*_2_*q_e_*^2^) + *t*/*q_e_* (8)

where *q_t_* is the adsorption capacity at any time. *K*_1_ and *K*_2_ are the adsorption rate constants.

From the correlation coefficient R^2^ values, the pseudo-second-order isotherm model described the adsorption processes well, as shown in Table 3. This model showed that the adsorption process was controlled by chemical adsorption processes related to the electrostatic interaction discussed in the previous section.

#### 3.2.4. FT-IR Analysis

**Figure 15:**
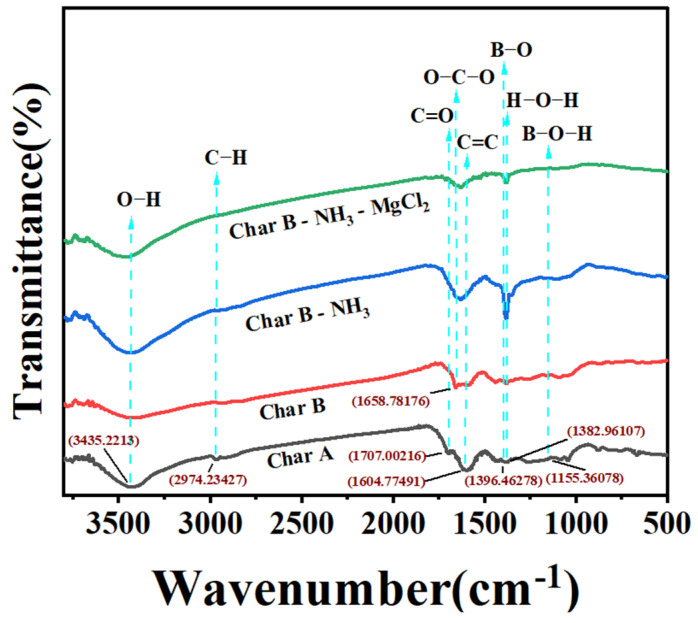
FT-IR spectra of char A and char B before boron adsorption and those of char B with NH_3_ nanobubbles and char B with 5 ppm MgCl_2_ and NH_3_ nanobubbles after boron adsorption at pH 9. (Adsorbent mass/volume of solution (m/V) is 0.4 g/L, measured temperature is 298 K, adsorption time is 2 h).

The chemical functional groups in char A and char B before boron adsorption, and in char B with NH_3_ nanobubbles and char B with 5 ppm MgCl_2_ and NH_3_ nanobubbles after boron adsorption at pH 9, were analyzed via FT-IR, and the results are shown in Figure 15. All adsorbents exhibited peaks at 3435 cm^−1^, 2974 cm^−1^, 1707 cm^−1^, 1658 cm^−1^, 1604 cm^−1^, and 1382 cm^−1^, which belonged to the stretching vibrations of O-H, C-H, C=O, O-C-O, C=C, and H-O-H [69,70,82,83,84,85]. The broad peaks in the 1396 cm^−1^ and 1155 cm^−1^ regions belonged to the stretching vibrations of B-O and B-O-H [86,87,88]. For char B with NH_3_ nanobubbles and char B with 5 ppm MgCl_2_ and NH_3_ nanobubbles, after adsorption, both adsorbents exhibited a peak at 1396 cm^−1^ corresponding to B-O stretching vibration, which was stronger than the corresponding peaks in char A and char B.

#### 3.2.5. XPS Analysis

**Figure 16:**
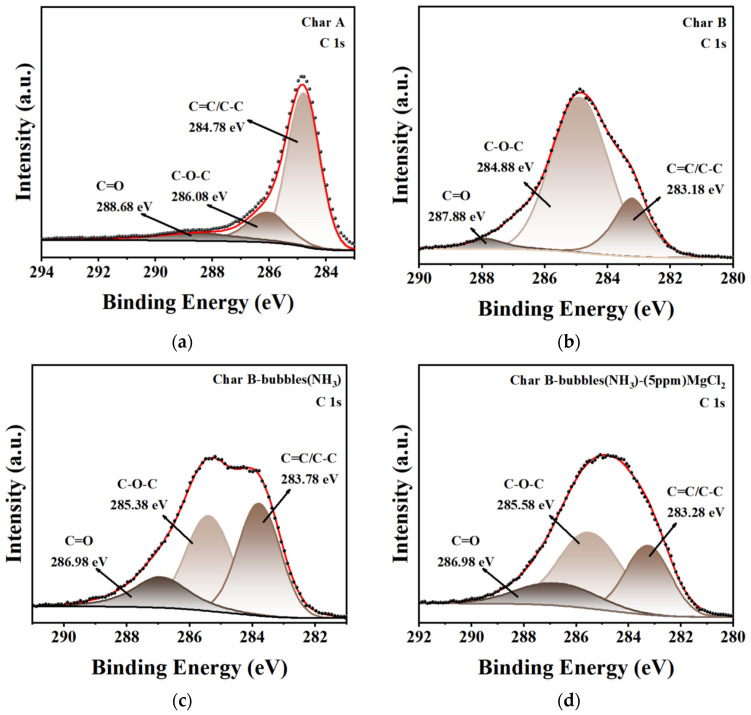

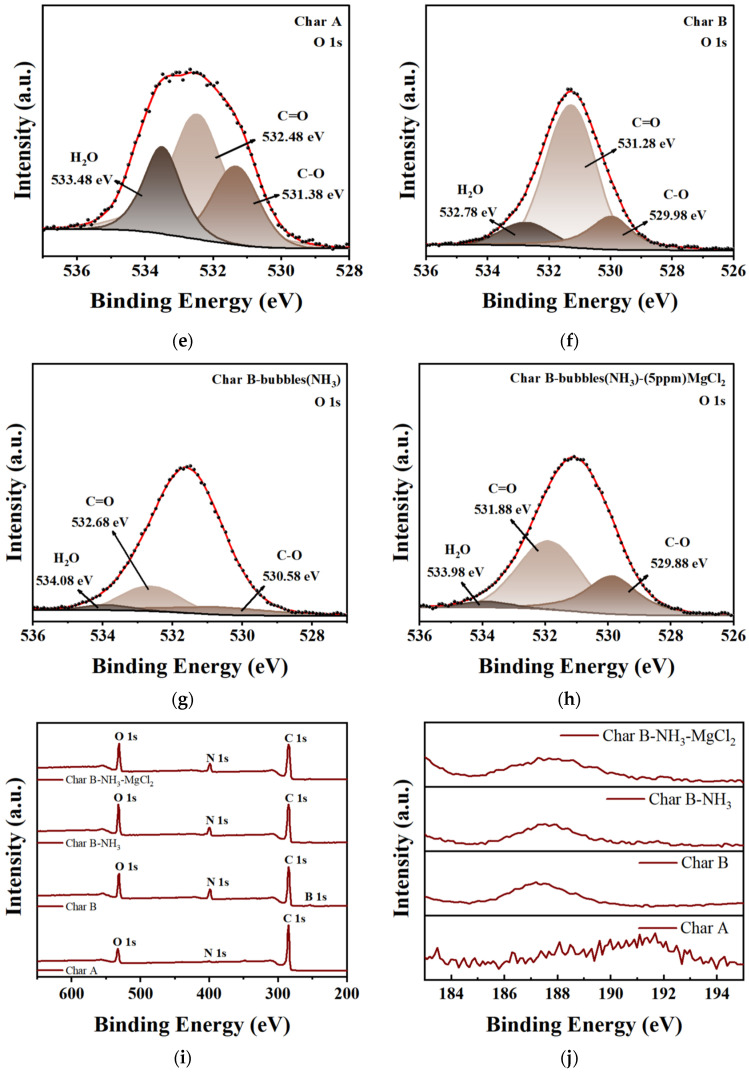
XPS spectra of char A and char B before boron adsorption and those of char B with NH_3_ nanobubbles and char B with 5 ppm MgCl_2_ and NH_3_ nanobubbles after boron adsorption at pH 9. (Adsorbent mass/volume of solution (m/V) is 0.4 g/L, measured temperature is 298 K, adsorption time is 2 h). (**a**–**d**) XPS spectra of C 1s of char A and char B before boron adsorption and those of char B with NH_3_ nanobubbles and char B with 5 ppm MgCl_2_ and NH_3_ nanobubbles after boron adsorption. (**e**–**h**) XPS spectra of O 1s of char A and char B before boron adsorption and those of char B with NH_3_ nanobubbles and char B with 5 ppm MgCl_2_ and NH_3_ nanobubbles after boron adsorption. (**i**) XPS survey scans of char A and char B before boron adsorption and those of char B with NH_3_ nanobubbles and char B with 5 ppm MgCl_2_ and NH_3_ nanobubbles after boron adsorption. (**j**) XPS spectra of B 1s of char A and char B before boron adsorption and those of char B with NH_3_ nanobubbles and char B with 5 ppm MgCl_2_ and NH_3_ nanobubbles after boron adsorption.

To obtain more detailed information about the functional groups on the surfaces of char A and char B before boron adsorption, and on char B with NH_3_ nanobubbles and char B with 5 ppm MgCl_2_ and NH_3_ nanobubbles after boron adsorption at pH 9, XPS survey spectra were obtained and are shown in Figure 16. The C 1s signals of char A had three characteristic peaks for C=C/C-C (284.78 eV), C-O-C (286.08 eV), and C=O (288.68 eV), as shown in Figure 16a. However, for char B before boron adsorption and the two types of char B after boron adsorption, the peaks of C=C/C-C, C-O-C, and C=O were red-shifted, as shown in Figure 16b, Figure 16c, and Figure 16d, respectively. The relative content of C=C/C-C in char B was much lower than that in char A, and the relative content of C=C/C-C in the two types of char B after boron adsorption was lower than that in char A. The same phenomena are shown in the peaks of C=O and C-O for O 1s signals in Figure 16e–h. These results imply that the surrounding chemical environment around the adsorbent species of C=C/C-C, C-O-C, and C=O had changed in the char B modified from char A and the two types of char B after boron adsorption. The XPS survey scans and the XPS spectra of B 1s are shown in Figure 16i,j. There was a relatively weak B peak between 187.1 eV and 187.5 eV [89], as shown in Figure 16j, which was consistent with the FT-IR analysis results.

#### 3.2.6. Removal of NH_4_^+^ Ions

**Figure 17:**
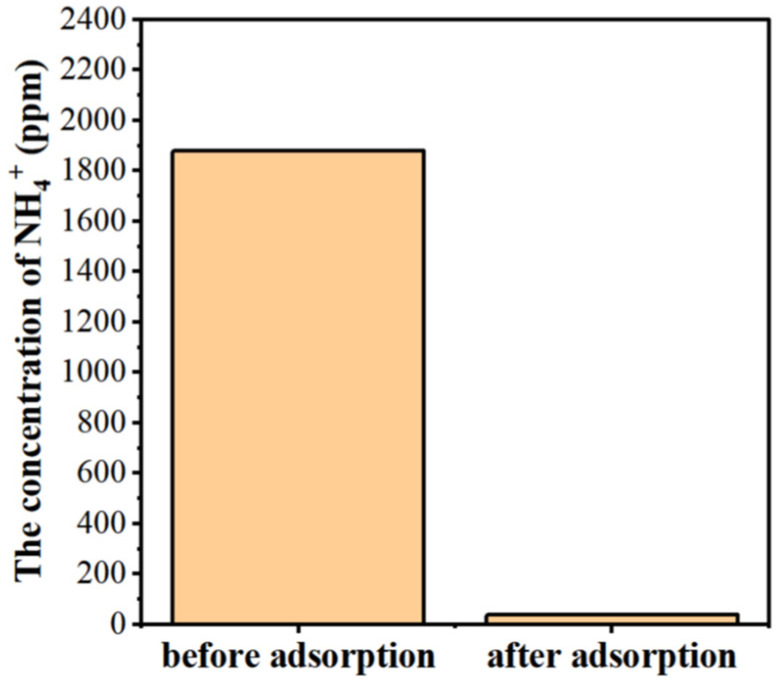
NH_4_^+^ ion removal using zeolite molecular sieves for adsorption and boiling.

In wastewater treatment, NH_4_^+^ ions, as indicated in Equations (2) and (3), must be removed from the aqueous solution after NH_3_ nanobubble and char treatment to remove boron. They were removed as NH_3_ gas, and in order to remove the NH_3_ gas, zeolite molecular sieves [90], which are used to adsorb NH_4_^+^ ions, were placed into the NH_4_^+^-containing aqueous solution and it was heated to boiling for one hour. The NH_4_^+^ removal effect is shown in Figure 17. Of the initial concentration of 1900 ppm of NH_4_^+^, less than 50 ppm remains after removal.

## 4. Conclusions

Sugarcane bagasse was heated at 400 °C to prepare char, and the char was modified using N, N-dimethylformamide, EDTA, and triethylamine. The aqueous solution was regulated at an alkaline pH, where the boron was mainly present as B(OH)_4_^−^ ions. If the adsorbent surface is positive, it is suitable for adsorption of B(OH)_4_^−^ anions. Therefore, the negative char surface was changed to positive by adding MgCl_2,_ and thus the B(OH)_4_^−^ ions could be adsorbed. Next, to increase the adsorption, NH_3_ nanobubbles (10% NH_3_ and 90% N_2_) with a large specific surface area were added to the aqueous solution. The nanobubble surface changed from slightly negative to positive with the addition of the Mg^2+^ ions. Therefore, the NH_3_ nanobubble surface containing Mg^2+^ and NH_4_^+^ ions could adsorb B(OH)_4_^−^ ions. The boron-adsorbed nanobubbles could coagulate the boron-modified char, removing more boron from the aqueous solution. The interaction between the NH_3_ nanobubbles and modified biochar was modeled using extended DLVO theory. Considering drinking water quality standards, the optimum conditions comprised 5 ppm MgCl_2_ addition at pH 9. The maximum boron adsorption capacity was 36 mg per 1 g char according to the Langmuir adsorption isotherm. The B-O stretching vibration was observed using FT-IR analysis after absorption on the modified char, and XPS spectra proved the boron was adsorbed on the modified char. After boron removal, ammonia was also removed using zeolite molecular sieves and heating.

## Figures and Tables

**Table 1:** Textural parameters of char A and char B.

Sample	BET Surface Area	Pore Diameter
	(m^2^/g)	(nm)
Char A	42.56	1.24
Char B	1.59	4.87

**Table 2:** Comparison of boron adsorption materials.

Adsorbent	Char B–MgCl_2_ (5 ppm)–NH_3_ Nanobubbles	Electrocoagulation [18]	Nylon-Based Chelating Fibers [31]
Adsorption capacity	36 mg/g	10 mg/g	12 mg/g

**Table 3:** Kinetic parameters of boron adsorption using pseudo-first-order and pseudo-second-order kinetic models.

**Pseudo-First-Order Model**		
***K*_1_ (min^−1^)**	***q_e_* (mg/g)**	**R^2^**
6.836 ± 1.187	35.06 ± 0.92	0.9757
**Pseudo-Second-Order Model**		
***K*_2_ (min^−1^)**	***q_e_* (mg/g)**	**R^2^**
4.061 ± 3.076	34.35 ± 3.08	0.6418

## Data Availability

The authors confirm that the data supporting the findings of this study are available within the article.
